# Individualized Prediction for Risk of Recurrence in Stage I/II Melanoma Patients With Negative Sentinel Lymph Node

**DOI:** 10.1002/cam4.70441

**Published:** 2024-11-29

**Authors:** Shu‐Ching Chang, Kristel Lourdault, Gary L. Grunkemeier, Douglas A. Hanes, Shih‐Ting Chiu, Stacey Stern, Richard Essner

**Affiliations:** ^1^ Providence St. Joseph Health Portland Oregon USA; ^2^ Providence Saint John's Cancer Institute Santa Monica California USA; ^3^ Oregon Health & Science University Portland Oregon USA

**Keywords:** Gompertz model, negative sentinel lymph node, risk of recurrence, stage I/II melanoma

## Abstract

**Background:**

Despite the favorable prognosis of AJCC stage I/II melanoma patients, up to 20%–30% will develop metastases. Our objective is to predict long‐term risk (probability) of recurrence in early‐stage melanoma patients.

**Methods:**

A Risk Score to predict long‐term recurrence was developed using Cox regression based on 2668 patients. Five clinicopathological risk factors were included. The association of the Risk Score with the risk of recurrence was evaluated using parametric models (exponential, Weibull, and Gompertz models) and compared to the Cox model using the Akaike information criterion. The discrimination of the model was measured by time‐dependent ROC analyses. A calibration curve was used to evaluate the agreement between predicted and observed recurrence probabilities.

**Results:**

The bootstrap adjusted C‐index was 0.76 (95% CI, 0.74–0.79) overall and 0.87 (0.83–0.90) and 0.82 (0.78–0.85) at one and two years, respectively, and then remained above 0.70 up to 20 years. The Gompertz model for prediction of survival from the Risk Score showed the best performance and displayed good agreement with the Kaplan–Meier curves. The calibration curve of the Gompertz model showed a good agreement between predicted and observed 2‐, 5‐, and 10‐year risk of recurrence. Population‐level analysis demonstrated a significant association of Risk Score with risk of recurrence, with 10‐year risks of recurrence of 4.5%, 13.0%, and 33.7% in the first, second, and third tertiles, respectively.

**Conclusion:**

We developed a Risk Score to predict long‐term risk of recurrence for early‐stage melanoma patients. A Gompertz survival model fit to the Risk Score allows for individualized prediction of time‐dependent recurrence risk.

## Graphical abstract

Up to 20%–30% of stage I/II melanoma patients will ultimately develop metastases. In this study, we developed a clinical Risk Score model to predict long‐term risk of recurrence.

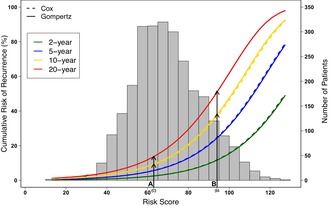

## 1 Introduction

Most new melanoma cases are diagnosed at early stages, 75% and 15% at AJCC stages I and II, respectively (Surveillance, Epidemiology, and End Results (SEER) Program Populations (1969–2018), National Cancer Institute 2019) [1]. Patient prognosis and risk of recurrence are commonly estimated based on the primary tumor characteristics (site, thickness, and ulceration status) and the sentinel lymph node (SLN) status. Unfortunately, individually, none of the clinical or pathological factors provides an accurate individual prognosis and risk assessment. The SLN status, considered as the most important predictive factor, remains highly inaccurate, as up to 20%–30% of tumor‐negative SLN (−SLN) patients will ultimately develop metastases [2, 3], with distant recurrence representing the most common type [4, 5, 6]. Despite the favorable prognosis of AJCC stage I/II melanoma patients with −SLN, the 5‐year recurrence‐free survival ranges from 76%–90% [7]. Furthermore, studies have shown that patients experiencing distant metastasis have worse overall survival compared to patients experiencing local or regional recurrences [8, 9].

Currently, there is no consensus on patients' follow‐up schedule and which blood or imaging exams AJCC stage I/II patients should have. As a result, these patients often require a long follow‐up. The development of targeted and immunotherapies has greatly improved stage III/IV melanoma patients' survival [10], and clinical trials suggest a survival benefit for stage IIB/C patients [11, 12]. However, these therapies have several side effects and should only be used for high‐risk patients. Thus, there is a need to develop tools to identify patients at the highest risk—who need therapy and will benefit from it; and those at the lowest risk—who may be spared the toxicities and expense of therapy.

Several clinical studies have identified risk factors associated with recurrence in early‐stage melanoma patients and developed models to predict recurrence [9, 13, 14, 15, 16, 17, 18], but none of them are predictive of long‐term recurrence. The objective of this study is to investigate the association between clinicopathological risk factors and risk (probability) of recurrence and to develop a Risk Score predicting long‐term risk of recurrence in AJCC stage I/II melanoma patients. We used parametric survival analyses [19], which allow for individualized long‐term prediction using a simple formula that may help physicians identify melanoma patients who have a higher risk of recurrence.

## 2 Methods

### 2.1 Patients and Data Collection

A total of 2668 AJCC stage I/II melanoma patients who had wide excision of their primary tumor and −SLN biopsy were identified from Multicenter Selective Lymphadenectomy Trial‐I (MSLT‐I) [20] and Saint John's Cancer Institute (SJCI) database and were included in our study. After wide excision of their primary tumor, patients were monitored by clinical exam, blood test, and radiographic imaging to detect recurrences. Recurrence was defined as the development of metastases in distant organs or nondistant sites, which includes in‐transit, local, nodal, or regional recurrence. For MSLT‐1, the follow‐up schedule was: every 3 months for the first 2 years, every 4 months during Year 3, every 6 months for Years 4 and 5, then annually until Year 10. Patients who received care at SJCI were followed according to the standard of care guidelines, that is, every 3–12 months depending on the stage for the first 5 years, then annually [21]. The time to recurrence was defined as the duration between the SLN biopsy and the detection of the first recurrence, typically by clinical exam or chest X‐ray. Clinical factors (age at diagnosis, sex) and tumor characteristics (Breslow thickness, site of primary tumor, ulceration status) were collected and recorded for all patients.

This study was approved through Providence Health and Services and eIRB protocols studies 2,020,000,522 and 2,019,000,139.

### 2.2 Statistical Analysis

Descriptive data, including patient demographics and tumor characteristics, were summarized by mean with standard deviation (SD) or median with interquartile range (IQR) for continuous variables and frequency with percentage for categorical data.

### 2.3 Development and Validation of the Risk Score

Five clinicopathological risk factors, including age at diagnosis, sex, ulceration status, site of primary tumor, and Breslow thickness, were selected based on a literature review, known clinically prognostic factors, and availability in the database. All were included in our final Cox regression model with no stepwise variable selection performed [22]. Risk of recurrence probabilities were calculated based on time from the date of SLN biopsy to the date of first recurrence (distant or nondistant) or death from melanoma if patients did not have recurrence (we assumed that patient deaths from melanoma were due to recurrence). Patients lost in follow‐up or that died from other causes were censored. The proportional hazards assumption was evaluated using statistical tests and graphical diagnostics based on the scaled Schoenfeld residuals using function “cox.zph” in R package “survival” [23]. The linearity of the continuous variables in relation to the risk of recurrence was assessed based on Martingale residuals using function “ggcoxfunctional” in R package “survminer” [24]. Breslow thickness was log2‐transformed to account for its nonlinear relationship with the risk of recurrence. The robustness of the final multivariable Cox regression model was evaluated with a mixed effects model, taking data sources as a random effect to account for within‐cluster homogeneity in outcomes [25, 26].

The final prediction model was then implemented in a point scoring system as a calculation tool for predicting the individualized risk of recurrence over time. To generate a simple point score, linear predictor values (the sum of predictor values times their predictor effects) were scaled and rounded to a Risk Score. Specifically, each risk factor was assigned a point ranging from 0 to 100, where the biggest impact factor is identified as a reference; the other factors are then assigned points based on their proportion to the biggest impact risk factor. The Risk Score for each patient was obtained by summing the assigned points for each prognostic risk predictor. Point scoring system and calibration plots were generated using the statistical R package rms [27].

The overall prediction performance of the model was validated for discrimination on the entire cohort using bootstrapping, the preferred approach for internal validation [26], with 1000 resamples [28]. Discrimination was measured by bootstrap‐adjusted Harrell's concordance index (C‐index) [29, 30] with a 95% confidence interval (CI). Time‐dependent areas under the receiver operating characteristic (ROC) curve (AUC) with 95% CIs were also estimated using R package survival ROC [31]. In general, an AUC of 0.5 suggests no discrimination, 0.7 to 0.8 is considered acceptable, 0.8 to 0.9 is considered excellent, and more than 0.9 is considered outstanding [32]. A calibration curve was used to evaluate the agreement between predicted and observed recurrence probabilities. To evaluate the generalizability of the model across two data sources, an internal–external cross‐validation was performed in which the model was fitted using data from one data source and validated in the other that was left out [26].

### 2.4 Application of Risk Score

We evaluated the association of the Risk Score and risk of recurrence using several types of survival models, including Cox proportional hazards, exponential, Weibull, and Gompertz models [33], and compared the model performance based on the Akaike Information Criterion (AIC). AIC is defined as AIC = −2 ln (L) + 2 k, where k is the number of model parameters and L is the maximum (partial) likelihood of the model (i.e., maximum partial likelihood for the Cox model; maximum likelihood for parametric models). AIC is a measure of goodness of fit for statistical models that deals with the tradeoff between bias and variance. Lower AIC values indicate a better model fit [34].

For population‐level analyses, based on their Risk Score, patients were stratified into 3 groups (tertiles) containing equal numbers and compared with respect to their risk of recurrence using Kaplan–Meier and Gompertz methods. Individualized predictions of 2‐, 5‐, 10‐ year as well as 20‐year risks of recurrence were developed and visualized. All tests were two‐sided, and statistical significance was set at *p* < 0.05. Statistical analyses were performed using the R software, version 4.1.2 (R Core Team 2022).

## 3 Results

### 3.1 Patient Characteristics

A total of 2668 AJCC stage I/II melanoma patients with −SLN were included in the final analysis cohort: 407 (15%) were patients from the MSLT‐I clinical trial [20] and 2261 (85%) were patients from the SJCI database. The median follow‐up time of the entire cohort was 9.2 years (IQR 6.0–11.6), with 10.0 years (IQR 7.1–10.0) and 8.9 years (IQR 5.9–12.3) for MSLT‐I and SJCI cohorts, respectively (Table 1). During the follow‐up period, 427 (16%) patients had recurrence, with first recurrence in nondistant sites (53.7%, *n* = 225), distant sites (42.8%, *n* = 183), or unknown/multiple sites (4.5%, *n* = 19). Among the 2668 patients in our cohort, 237 (8.9%) died from melanoma and 311 (12%) from non‐melanoma causes. In the entire cohort, patients had a mean age of 55 years (SD, 15.9) and median Breslow thickness of 1.2 mm (IQR, 0.8–1.9). The majority of patients were male (59.7%, *n* = 1593), had primary tumors without ulceration (78.6%, *n* = 2096), and had tumors located on the extremities (42.3%, *n* = 1129) or trunk (37.7%, *n* = 1005) (Table 1).

**TABLE 1:** Patient characteristics.

	All *n* = 2668	MSLT‐I *n* = 407 (15.3%)	SJCI *n* = 2261 (84.7%)
Age (years)			
Mean (SD)	54.7 (15.9)	52.9 (13.3)	55 (16.3)
Sex, *n* (%)			
Female	1075 (40.3)	168 (41.3)	907 (40.1)
Male	1593 (59.7)	239 (58.7)	1354 (59.9)
Ulceration, *n* (%)			
Absent	2096 (78.6)	278 (68.3)	1818 (80.4)
Present	434 (16.3)	114 (28.0)	320 (14.2)
Unknown	138 (5.2)	15 (3.7)	123 (5.4)
Primary site, *n* (%)			
Extremity	1129 (42.3)	199 (48.9)	930 (41.1)
Head/neck	534 (20.0)	74 (18.2)	460 (20.3)
Trunk	1005 (37.7)	134 (32.9)	871 (38.5)
Breslow thickness (mm)			
Median (Q1, Q3)	1.2 (0.8, 1.9)	1.7 (1.3, 2.6)	1.0 (0.7, 1.7)
AJCC stage, *n* (%)			
IA	1017 (40.0)	13 (3.3)	1004 (47.0)
IB	772 (31.0)	181 (46.0)	591 (28.0)
IIA	396 (16.0)	94 (24.0)	302 (14.0)
IIB	259 (10.0)	86 (22.0)	173 (8.1)
IIC	78 (3.1)	18 (4.6)	60 (2.8)
Unknown	146	15	131
Follow‐up (years)			
Median (Q1, Q3)	9.2 (6.0, 11.6)	10.0 (7.1, 10.0)	8.9 (5.9, 12.3)
Maximum	28.2	14.6	28.2
Total patient years	25,080	3375	21,705

Abbreviations: MSLT‐I, Multicenter Selective Lymphadenectomy Trial‐I; SD, Standard Deviation; SJCI, Saint John's Cancer Institute.

### 3.2 Factors Predictive of Recurrence

Table 2 shows the results of the final Cox regression model. The factors associated with increased risk of recurrence were increasing Breslow thickness (2‐fold change, hazard ratio [HR], 1.89; 95% CI, 1.72–2.07), primary tumor located on head and neck region (HR, 1.64; 95% CI, 1.28–2.10), presence of ulceration (HR, 1.51; 95% CI, 1.20–1.90), and age at diagnosis (HR, 1.01; 95% CI, 1.00–1.02). The estimates from the Cox model showed very similar estimates (the HR and 95% CI to the precision of 2 decimal places were matched) using a mixed effects Cox regression model, accounting for clustering effects of two different data sources.

**TABLE 2:** Hazard ratios (95% CI), along with scoring system points, based on the final Cox model. The risk score for a given patient is obtained by summing the points for each risk factor.

Risk factors	Hazard ratio^a^ (95% CI)	Points assigned
Age (years)	1.01 (1.00–1.02)	Age × 0.21
Sex		
Female	Reference	+0
Male	1.04 (0.84–1.29)	+1
Ulceration		
Absent	Reference	+0
Present	1.51 (1.20–1.90)	+8
Unknown	2.05 (1.48–2.85)	+14
Primary site		
Extremity	Reference	+0
Head/neck	1.64 (1.28–2.10)	+10
Trunk	1.13 (0.89–1.43)	+2
Breslow (mm)	1.89 (1.72–2.07)	log_2_ (Breslow) × 12.8 + 49.0

^a^ Hazard ratio, the exponential of the coefficients of Age (year), Sex, Ulceration, Primary Site, and log_2_(Breslow) in the Cox model.

### 3.3 Prediction for Risk of Recurrence

The Risk Score was constructed based on the fitted coefficients of 5 clinicopathological factors from the final Cox model (i.e., log(HR)). The points assigned to each risk factor were as follows: Breslow thickness (mm, log2 scale) was assigned points ranging from 0 to 100 for its biggest impact on risk of recurrence (i.e., absolute maximum value of its coefficient, log(HR), multiplied by the value range of the log2‐scale Breslow) and was identified as a reference. The other factors were then assigned points based on their proportion to Breslow thickness. The Risk Score for each patient is then obtained by summing the assigned points for each risk factor (Table 2) [27].

**FIGURE 1:**
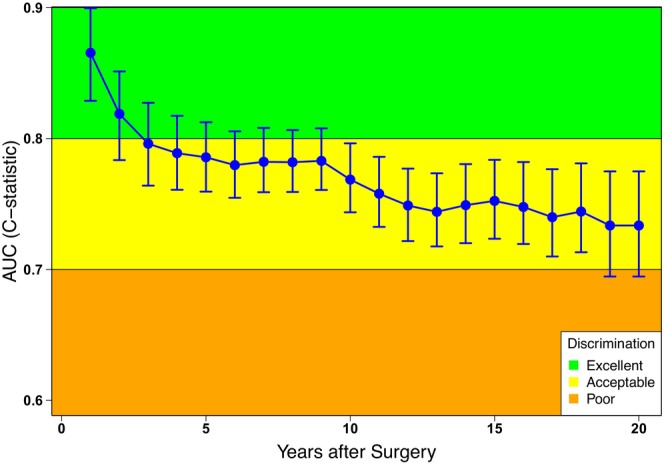
Time‐dependent AUC with 95% CIs based on the Cox model for risk of recurrence using time‐dependent ROC analysis.

The bootstrap‐adjusted C‐index of the developed Risk Score was 0.76 (95% CI, 0.74, 0.79) overall. The time‐dependent ROC curves achieved AUC values of 0.87 (95% CI, 0.83–0.90) and 0.82 (95% CI, 0.78–0.85) at the first and second follow‐up years, respectively, and then remained above 0.7 out to 20 years (Figure 1). In internal–external cross‐validation, the C‐index for a model based on one data source and applied to the other that was left out ranged from 0.67 (developed on SJCI and validated on MSLT‐I) to 0.74 (developed on MSLT‐I and validated on SJCI).

**FIGURE 2:**
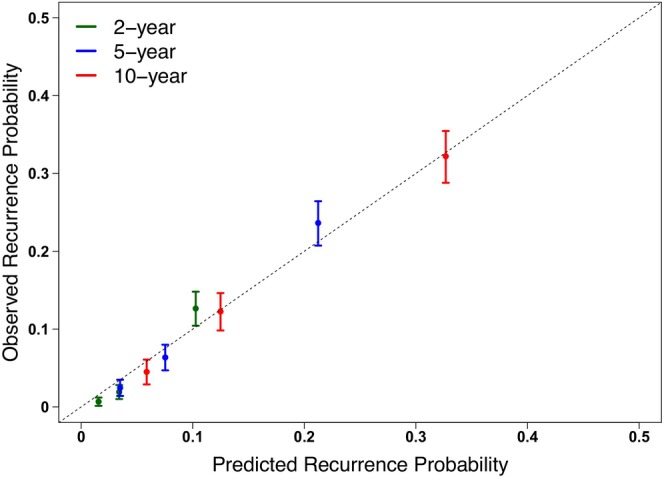
Calibration curve with 95% CIs comparing observed (actual) and predicted recurrence probabilities for the entire study cohort. The dotted gray line indicates a perfect calibration model in which the predicted recurrence probabilities are identical to the actual recurrence proportions.

**FIGURE 3:**
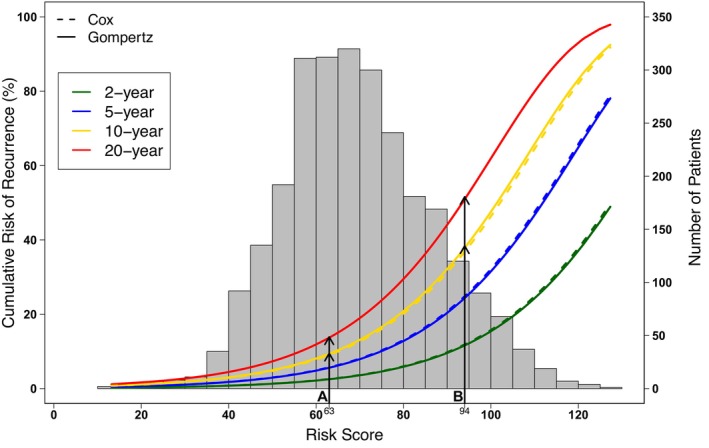
The curves represent the predicted risk of recurrence (%) determined by the Cox model (dashed lines) or the Gompertz model (solid lines) at 2, 5, 10 and 20 years in green, blue, yellow, and red, respectively. The histogram refers to the Risk Score distribution in the study cohort; each bar indicates the number of patients with that specific Risk Score.

By comparing the performance of survival models for the association of Risk Score and risk of recurrence, we determined that the Gompertz model was the best fit with the lowest AIC of 3893.7 (Cox, 6062.1; exponential, 3924.4; Weibull, 3914.6) and displayed good agreement with the Kaplan–Meier curves. The calibration curve of the model showed a good agreement between predicted and observed 2‐, 5‐, and 10‐year risk of recurrence (Figure 2). The Gompertz model for the prediction of risk of recurrence by the patient's Risk Score is presented in Figure 3, with a formula given in the Appendix S1 and Calculator. The risk of recurrence probabilities using the Gompertz method showed strong agreement with those using the Cox model during the period of observation (Figure 3).

**FIGURE 4:**
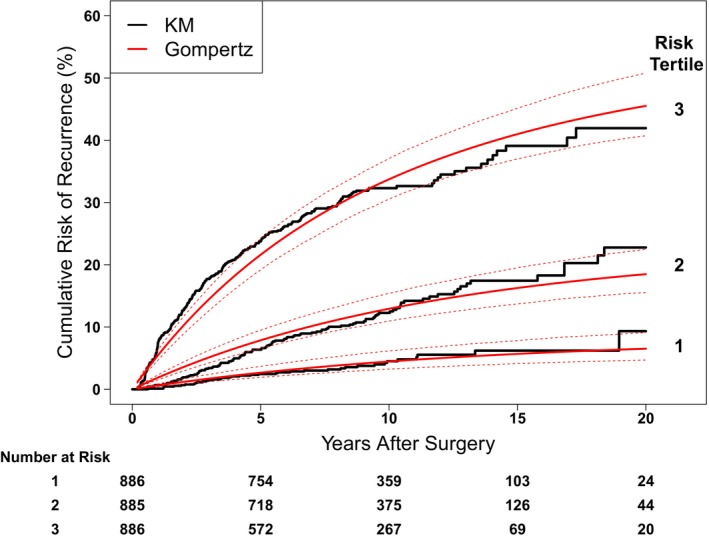
Risk of recurrence over time, stratified by Risk Score tertiles using Kaplan–Meier method in black and the fitted Gompertz curves in red. Dashed lines show 95% CIs of the fitted Gompertz curves.

For population‐level analyses, the Risk Scores of all 2668 patients were calculated, and patients were stratified into 3 groups (tertiles) based on their Risk Score: low‐risk score (13–62), intermediate‐risk score (62–76), and high‐risk score (76–127), and compared. There was a significant difference in risk of recurrence between Risk Score tertiles, with 10‐year risk of recurrence of 4.5% (3.2–6.1), 13.0% (11.1–15.3), and 33.7% (30.2–37.1) in the first, second, and third tertiles, respectively (Figure 4). Gompertz curves were well matched with the Kaplan–Meier curves for each tertile.

### 3.4 Examples of Individualized Prediction for Risk of Recurrence Using the Point Scoring System

To illustrate how to use the point scoring system, we created two hypothetical patients with different clinical and pathological factors as described in Table 3. Patient A is a 35‐year‐old male with a 1.2 mm thick primary melanoma located on the truck. Patient B is a 70‐year‐old male with a 1.8 mm thick ulcerated primary melanoma located on the head/neck region. The Risk Score for each patient was calculated by adding the points assigned for each of their 5 risk factors as described in Table 2, resulting in a Risk Score of 63 for patient A and 94 for patient B (Table 3). The risk of recurrence of a patient at year Y can be determined by using the formula given in the Appendix S1 and Calculator (Table S1). Alternatively, using Figure 3, the risk of recurrence for an individual patient for a certain time point can be determined by reading the individual's Risk Score on the x‐axis and the corresponding risk of recurrence probability on the y‐axis according to the year of prediction. Thus, patient A's and B's predicted risk of recurrence, using the Gompertz model, are 9.5% and 38.4% at 10 years, and 13.9% and 51.6% at 20 years, respectively (Figure 3 and Table 3). This demonstrates the increased amount of information available by using a parametric model for survival—survival prediction beyond the follow‐up year.

**TABLE 3:** Clinical and pathological risk factors of the two hypothetical patients A and B, with their corresponding Risk Score, and risk of recurrence at 2, 5, 10, and 20 years.

Risk factors	A	Points	B	Points
Age (years)	35	7.4	70	14.7
Sex	Male	1	Male	1
Ulceration	Absent	0	Present	8
Primary Site	Trunk	2	Head/neck	10
Breslow (mm)	1.2	52.4	1.8	59.9
Risk Score		63		94
Risk of Recurrence probability, %				
Cox model				
2 years		2.7		11.8
5 years		6.0		24.7
10 years		9.4		36.6
Gompertz model				
2 years		2.6		11.8
5 years		5.7		24.8
10 years		9.5		38.4
20 years		13.9		51.6

## 4 Discussion

Sentinel lymph node biopsy is an integral part of stage I/II melanoma patients' standard of care and is used as a staging and prognostic tool. Although most patients with −SLN have a low risk of recurrence and a good prognosis, some of them will develop metastases [2, 3]. To investigate the association of risk factors with risk of recurrence, we developed a Risk Score to predict long‐term risk of recurrence with the aim of helping clinicians better understand individual patients' risk of recurrence and possibly lead to a more standardized approach.

Our Risk Score prediction model includes 5 clinical and pathological factors: age at diagnosis, sex, Breslow thickness, site and ulceration status of the primary tumor. We used the AIC to compare the relative performance of several survival models including Cox, exponential, Weibull, and Gompertz models, for the association of Risk Score and risk of recurrence, and demonstrated that the Gompertz model showed the best performance for its lowest AIC. Although it is not as popular as the semiparametric Cox proportional‐hazards model, the Gompertz distribution is a powerful and well‐suited model for survival and can be justified by invoking some biological reasoning—that the death rates increase exponentially with age—which has been shown to be more accurate, especially in the older population [32, 35, 36, 37]. Non‐melanoma death was censored in our risk models, but the competing risk analyses [38, 39], taking non‐melanoma death as the competing risk, resulted in risk estimates that were only 0.4% and 1.9% smaller at 10 years and 20 years, respectively. To evaluate the robustness of the final Cox model on the pooled data of two data sources, a mixed effects Cox regression model takes data sources as a random effect to account for within‐cluster homogeneity in outcomes. In addition, internal–external validation was performed to further evaluate the generalizability of predictions of the model [18, 26].

The main strengths of this study include the large cohort size (2668 patients) with a very long and standardized follow‐up protocol and the ability to predict long‐term risk of recurrence. In addition, the overall discrimination performance of the model achieved a C‐index of 0.76 based on bootstrapping approach, which was a well‐established and preferred statistical technique for internal validation. Time‐dependent ROC analyses showed AUC values (95% CI) of 0.87 (0.83–0.90) and 0.82 (0.78–0.85) at one‐ and 2‐year follow‐up, respectively, and then remained above 0.70 out to 20 years. Furthermore, the prediction models were validated using the recommended internal–external validation procedure, which showed that the C‐index for a model based on one data source and applied to the other that was left out ranged from 0.67 to 0.74.

The 5 clinical and pathological factors used in the model are the most common factors recorded by physicians, which suggests that the point scoring system can be broadly used. Furthermore, as we demonstrated in this study, the point scoring system is easy to use and can be applied to stage I/II melanoma patients with −SLN. To our knowledge, this is one of the rare studies that focuses on the risk of recurrence in early‐stage melanoma patients. Recently, Risk Score models that predict the 5‐year risk of recurrence have been developed by Verver et al. and Stassen et al. [17, 18]. Our Risk Score model has the unique advantage that it can determine the risk of recurrence at different time points, such as 2, 5, 10, and even 20 years by using the Gompertz method, with an acceptable overall C‐index of 0.76 [32]. To fully capture the effect of patient Risk Score on recurrence risk, we take Risk Score as a continuous variable, allowing for predictions based on an individual Risk Score. Additionally, we stratify patients into low‐risk score (13–62), intermediate‐risk score (62–76), and high‐risk score (76–127) categories based on total Risk Score tertiles for comparison. Our analysis shows a significant difference in recurrence risk among the three groups, with 10‐year risk of recurrence of 4.5% (3.2–6.1), 13.0% (11.1–15.3) and 33.7% (30.2–37.1) for patients with low, intermediate, and high‐risk scores, respectively (Figure 4). This demonstrates that the risk of recurrence is effectively stratified, which may help with future treatment decisions and trial design [40, 41].

There are some limitations to this study that should be considered. First, it is a retrospective study, which has inherent selection biases. While results of a retrospective cohort cannot supplant results from a well‐planned prospective study, these data can help to further understand a topic in which the prospectively collected data is incomplete or unavailable, namely which patient risk factors are associated with increased risk of recurrence in early‐stage melanoma patients. Second, although the final model was internally validated as well as validated using the recommended internal‐external method [26], it was not externally validated on the fully independent external dataset. Therefore, we are planning on validating our risk model with an external cohort, when available, and also hopefully acquire prospective external validation in the future. Third, our model only predicts for any type of first recurrence, so it will be worthwhile for future study to develop a multistate model that can predict not only the risk of first recurrence but for a specific type of recurrence (e.g., in a distant organ) and also for transition probability for patients who developed other recurrences after their first recurrence over the course of the follow‐up period [42].

In summary, we created a point scoring system using 5 clinical and pathological factors that can accurately predict the risk of recurrence at different timepoints in AJCC stage I/II melanoma patients with −SLN. This easy‐to‐use point scoring system, specifically designed for patients with confirmed −SLN, can potentially help clinicians better understand individual patients' risk of recurrence. It could also help physicians adjust patients’ follow‐up schedule and treatment decision‐making. Indeed, in patients with high risk of recurrence, the frequency of follow‐up appointments could be increased, for instance, from every 6 months to every 3 months, and an early start of systemic therapy (targeted or immunotherapy) could also be recommended. On the other hand, patients with low risk of recurrence could be seen less often and spare them from stress and anxiety due to uncertain prognosis. This will ultimately improve the patient's survival. Also, with the increasing use of adjuvant immunotherapy for early‐stage melanoma patients, it would be highly valuable to develop an individualized point‐of‐care tool to predict the survival benefit obtained from the addition of such treatments and assess the impact of patient Risk Score on surveillance or potentially identify which patients would benefit from adjuvant immunotherapy.

## Author Contributions

**Shu‐Ching Chang:** conceptualization (lead), formal analysis (lead), investigation (lead), methodology (lead), writing – original draft (lead). **Kristel Lourdault:** conceptualization (lead), data curation (lead), investigation (lead), methodology (equal), writing – original draft (lead). **Gary L. Grunkemeier:** conceptualization (lead), formal analysis (equal), investigation (lead), methodology (lead), writing – review and editing (lead). **Douglas A. Hanes:** conceptualization (equal), methodology (equal), writing – review and editing (equal). **Shih‐Ting Chiu:** conceptualization (equal), methodology (equal), writing – review and editing (equal). **Stacey Stern:** conceptualization (equal), data curation (equal), writing – review and editing (equal). **Richard Essner:** conceptualization (lead), funding acquisition (lead), investigation (lead), writing – review and editing (equal).

## Ethics Statement

This study was approved through Providence Health and Services and eIRB protocols studies 2,020,000,522 and 2,019,000,139.

## Consent

Informed consent was obtained from all individual participants included in the study. Patients signed informed consent regarding publishing their data.

## Conflicts of Interest

Dr. Essner is on the Scientific Advisor Board and speaker's bureau for Castle Biosciences. The other authors have no relevant financial or nonfinancial interests to disclose.

## Supporting information


Table S1.

Appendix S1.

## Data Availability

The datasets generated during and/or analyzed during the current study may be shared with approved individuals upon request and through a written agreement with the authors.
