# Supplementary material for: Individualized Prediction for Risk of Recurrence in Stage I/II Melanoma Patients With Negative Sentinel Lymph Node
Source: Cancer Med. 2024 Nov 29;13(23):e70441. doi: 10.1002/cam4.70441 (PMC11605731; doi:10.1002/cam4.70441)
Supplement: Supplementary file 2 — Appendix S1. [file CAM4-13-e70441-s001.docx]

**APPENDIX**

The probability (risk) of recurrence at Y years after surgery for a patient with a Risk Score of RS is:

P(Recurrence| Y, a, b) = 1 - exp(-b*(exp(a*Y)-1)/a),

where a = -0.070 and b = exp(-7.491 + 0.051*RS).
